# Adaptation and validation of a quantitative *vanA*/*vanB* DNA screening assay on a high-throughput PCR system

**DOI:** 10.1038/s41598-024-54037-5

**Published:** 2024-02-12

**Authors:** Katja Giersch, Konstantin Tanida, Anna Both, Dominik Nörz, Denise Heim, Holger Rohde, Martin Aepfelbacher, Marc Lütgehetmann

**Affiliations:** Institute of Medical Microbiology, Virology and Hygiene, University Medical Centre Hamburg-Eppendorf (UKE), Martinistraße 52, 20246 Hamburg, Germany

**Keywords:** VRE, Vancomycin-resistant enterococci, Real time polymerase chain reaction, cobas6800, Molecular diagnostics, Bacterial infection, Diagnosis

## Abstract

Vancomycin resistant enterococci (VRE) are a leading cause of ICU-acquired bloodstream infections in Europe. The bacterial load in enteral colonization may be associated with a higher probability of transmission. Here, we aimed to establish a quantitative *vanA/vanB* DNA real-time PCR assay on a high-throughput system. Limits of detection (LOD), linear range and precision were determined using serial bacterial dilutions. LOD was 46.9 digital copies (dcp)/ml for *vanA* and 60.8 dcp/ml for *vanB*. The assay showed excellent linearity between 4.7 × 10^1^ and 3.5 × 10^5^ dcp/ml (*vanA*) and 6.7 × 10^2^ and 6.7 × 10^5^ dcp/ml (*vanB*). Sensitivity was 100% for *vanA* and *vanB*, with high positive predictive value (PPV) for *vanA* (100%), but lower PPV for *vanB* (34.6%) likely due to the presence of *vanB* DNA positive anerobic bacteria in rectal swabs. Using the assay on enriched VRE broth *vanB* PPV increased to 87.2%. Quantification revealed median 2.0 × 10^4^ dcp/ml in PCR positive but VRE culture negative samples and median 9.1 × 10^4^ dcp/ml in VRE culture positive patients (maximum: 10^7^ dcp/ml). The automated *vanA/B*_UTC assay can be used for *vanA/vanB* detection and quantification in different diagnostic settings and may support future clinical studies assessing the impact of bacterial load on risk of infection and transmission.

## Introduction

Over the last decades, vancomycin-resistant enterococci (VRE) have become a pathogen of concern for public health worldwide and were declared a pathogen with high priority in the global priority list of antibiotic-resistant bacteria by the World Health Organization (WHO)^1–3^. VRE are a major cause of hospital-acquired bloodstream infections in Europe and lead to higher mortality rates, length of stay and hospital costs compared to vancomycin-susceptible enterococci^4–10^. In the EU/EEA, vancomycin-resistance of invasive *Enterococcus faecium* isolates rose from 10.5% in 2015 to 18.3% in 2019^11^. Especially in patients after hematopoietic stem cell transplantation, VRE blood stream infections showed an association with lower overall survival and non-relapse mortality^12^. However, overall colonization is common and infection is relatively rare with mostly immunosuppressed and ICU patients at risk^13–15^. Recently, it has been shown that discontinuation of contact precautions for VRE and active VRE screening programs in Ontario hospitals led to an increase in VRE bloodstream infections^16^. On the other hand, contact isolation measures may also have a detrimental effect on patient outcomes^17,18^. Thus, there is an on-going debate across institutions, how to best identify patients at risk of infection or spreading of VRE, while preventing unnecessary isolation measures. Patient-related risk factors for infection and the dynamics of hospital spread are still a subject of research and it has been hypothesized that the respective enteric bacterial load of VRE may play a role in both infection and transmission^19^. Currently, *vanA* and *vanB* can be detected by qPCR using manual tests on a Light Cycler^20,21^ or automated tests on the BD MAX^22^ or Xpert Xpress systems^23^. Fully-automated PCR systems offer several advantages such as reproducibility, a lower risk of contamination and less hands-on time compared to manual PCR workflows. However, there is currently no qPCR assay that can be used on an easily scalable, high-throughput platform. Therefore, we here provide a tool to easily and reliably detect and quantify VRE bacterial loads in rectal swabs by real-time PCR of the *vanA* and *vanB* determinants on the Utility Channel (UTC) of the high-throughput cobas 5800/6800/8800 PCR systems. The cobas 5800/6800/8800 systems can measure more than 5000 samples per day and the use of CE-IVD reagents allow the implementation of laboratory-developed tests (LDT) compliant e.g. with the European Union In Vitro Diagnostic Medical Device Regulation (EU IVDR) in patient diagnostics.

## Material and methods

### *vanA/B*_UTC design and setup

**Figure 1:**
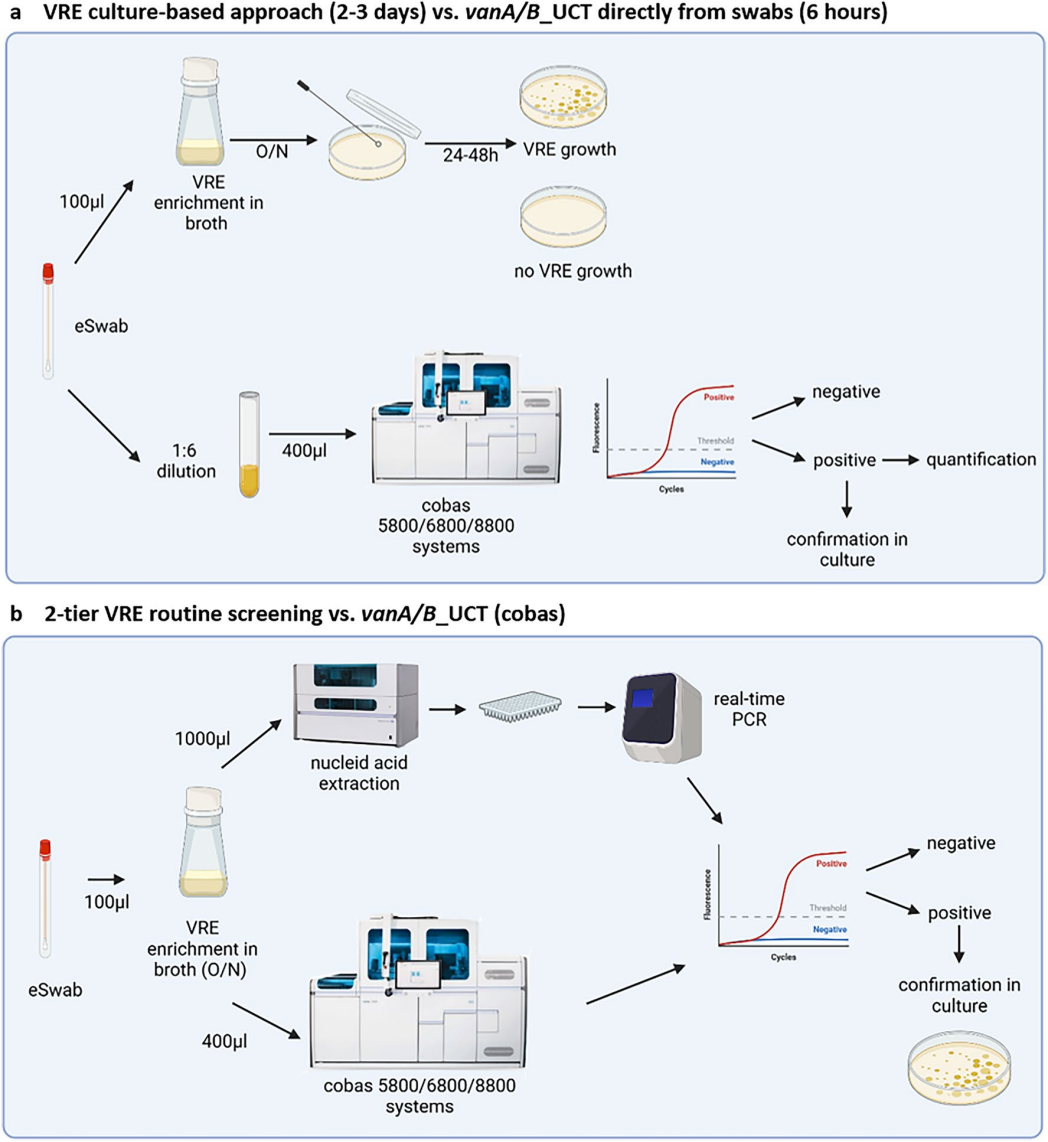
Workflow of the vanA/B_UTC assay using cobas 5800/6800/8800 systems (**a**) and the two-tier routine VRE screening compared to the two-tier vanA/B_UCT (**b**).

Previous published primer/probe-sets detecting *vanA* and *vanB* genes were selected^24^ and adapted for the cobas omni Utility Channel (UTC) chemistry (Roche, Mannheim, Germany) (Fig. 1a).

Primers were modified with 2′-O-methyl bases to prevent formation of primer dimers. For optimal melting temperature and binding stability, probes were conjugated to a minor groove binder at the 3′-end. All oligos used in this study are listed in Table 1. In brief, 29.3 µl of each primer stock solution (500 µM concentration) and 36.7 µl of each probe stock solution (100 µM concentration) was combined with 10 ml MMR2 in a 15 ml Falcon tube, mixed rigorously (10 min rolling) and added to the cobas omni Utility Channel cassettes according the manufacture’s recommendations. The cobas omni channel comes with a spike-in RNA full-process control, which is added automatically during extraction and is detected in channel 5 (see Table 2 for the full run protocol). Primers and probes were custom-made by Ella Biotech (Fuerstenfeldbruck, Germany) and Biomers (Ulm, Germany), respectively. This LDT *vanA/vanB* qPCR assay is henceforth referred to as *vanA/B*_UTC assay.

**Table 1:** Primer and probe sequences of the *vanA/vanB* duplex assay are listed.

Oligo type	Oligo name	Sequence 5′–3′	Final concentration in the reaction (nM)
Primers	vanA-FO	AGT CAA TAC TCT GCC CGG TT(OMe-U)	400
	vanA-RO	GCA GCG GCC ATC ATA C(OMe-G)	400
	vanB-FO	TCC GGT CGA GGA AC(OMe-G) AAA	400
	vanB-RO	GCC CTC TGC ATC CAA G(OMe-C)A	400
Probes	vanA-FAMmgb	FAM-CGT CAT ACA GTC GTT ATC-MGB-BMNQ535	100
	vanB-VICmgb	VIC-ACG GCA AAG AAA GTA TAT C-MGB-BMNQ535	100

Sequences are derived from a previously published assay by Fang et al.^24^. Indicated concentrations refer to the final oligo concentrations within the reaction mix. 2′O-methyl-RNA bases are indicated as “OMe-X”.

**Table 2:** Cobas omni Utility Channel run protocol for the vanA/B_UTC assay with internal control (IC).

Software settings					
Sample type	eSwab (400 µL)				
Channels	1: Not used	2: vanA	3: vanB	4: Not used	5: IC
RFI		2	2		2

PCR cycling conditions					
	UNG incubation	Pre-PCR step	1st measurement	2nd measurement	Cooling
No. of cycles	Predefined	1	5	45	Predefined
No. of steps		3	2	2	
Temperature		55 °C; 60 °C; 65 °C	95 °C; 55 °C	91 °C; 58 °C	
Hold time		120 s; 360 s; 240 s	5 s; 30 s	5 s; 25 s	
Data acquisition		None	End of each cycle	End of each cycle	

RFI (relative fluorescence increase) thresholds in channel 2 (vanA) and channel 3 (vanB) are used for automated result calls.

### Evaluation of analytical performance

Technical performance evaluation for the *vanA/B*_UTC assay was performed according to new EU regulations (2017/746 EU IVDR). Vancomycin-resistant *E. faecium* SX6010 (*vanA*) obtained from a clinical sample and *E. faecalis* ATCC 51299 (*vanB*) were used as reference strains in this study. To obtain a quantitative *vanA* and *vanB* standard, nucleic acids from these strains were purified using a MagNA-pure96 extractor (Roche diagnostics, Rotkreuz, Switzerland) and analysed on the Biorad QX100 Droplet Digital PCR System (Biorad, Hercules, California, USA). The unit of the standard is digital copies/ml (dcp/ml).

Lower limit of detection (LoD) was determined by serial two-fold dilution of *vanA-* and *vanB*-containing suspension (SX6010 and ATCC 51299) in Amies Transport Medium (Copan eSwab, Murrieta, CA, USA) ranging from 3452.5 dcp/ml to 6.9 dcp/ml (*vanA*) and 2736.0 dcp/ml, to 5.3 dcp/ml (*vanB*). N = 20 per dilution step and dilution was prepared using a Hamilton IVD STARlet liquid handler (Hamilton, Bonaduz, Switzerland). Linearity was assessed by tenfold serial dilution of *vanA-* and *vanB*-containing suspension (n = 3 per dilution step) between concentrations of approximately 5 × 10^1^ dcp/ml and 5 × 10^5^ dcp/ml. Linearity was calculated using Validation Manager software (Finbiosoft, Espoo, Finland).

The intra-run and inter-run precision was determined using three different 1:10 dilutions of the *vanA* and *vanB* reference isolate in triplicates on three different days. Within-laboratory precision was calculated as sum of squares of precision components. Precision was calculated as standard deviation (SD) with coefficient of variation (CV %) according to ANOVA statistics using Validation Manager (Finbiosoft).

A cross-reactivity study was performed using 47 isolates of 36 different common enteric bacteria including other enterococci (e.g., *E. avium, E. gallinarum, E. casseliflavus)* and samples from an external quality assessment were measured using the *vanA/B*_UTC assay.

### Routine VRE screening

Routine VRE screening at University Medical Centre Hamburg-Eppendorf, Germany, consists of a two-tier approach combining culture-based and molecular techniques (Fig. 1b). Briefly, 100 µl of Amies medium from the rectal swabs (eSwab, Copan) were transferred to 2 ml of VRE enrichment broth (Oxoid, Basinstoke, UK) and incubated over-night. Enriched broth was plated on ChromID VRE agar (bioMérieux, Marcy l’Etoile, France) and incubated at 37 °C for 24 to 48 h. After colonies displaying morphology typical of VRE on the VRE agar was detected, nucleic acids were extracted using the MagNA-pure96 system (Roche) with 200 µl extraction volume according to manufacturer’s recommendation and further analysed by qPCR on the LightCycler 480 II (Roche) using *vanA* and *vanB* specific primers and probes^24^. Simultaneously, colonies were identified by matrix-assisted laser desorption/ionization time-of-flight (MALDI-TOF, Bruker, Billerica, MA, U.S.A.) and automated susceptibility testing on a Vitek2 instrument (Biomérieux, Marcy-l’Etoile, France).

### Inclusivity and exclusivity testing

28 samples of an external quality assessment for VRE (INSTAND, Düsseldorf) were tested, which included 10 *vanA* and 7 *vanB* positive samples. For empirical exclusivity testing, a set of 47 different isolates, which consisted of 36 different gram-positive and gram-negative enteric bacteria, was applied.

### Clinical evaluation—directly on rectal swabs without enrichment: comparison of *vanA/B*_UTC to Xpert* vanA/vanB* and culture

For clinical validation, 196 rectal swabs from routine screening were diluted 1:6 with cobas PCR medium (Roche), directly measured using cobas 5800/6800/8800 systems and compared to culture and the CE-IVD cartridge-based Xpert *vanA/vanB* assay (Cepheid, Sunnyvale, CA, USA).

### Clinical evaluation—two-tier approach: comparison of *vanA/B*_UTC to routine VRE screening

In a second clinical validation the presence of *vanA/B* was determined in 374 rectal swabs samples using our routine VRE screening (see above) and compared to a similar two-tier workflow using the *vanA/B*_UTC assay. The rectal swabs that were detected positive using the VRE routine screening (181/374) were also diluted 1:2 with cobas PCR medium (Roche) and directly measured and quantified using the cobas 5800/6800/8800 systems.

The study was conducted according to the guidelines of the Declaration of Helsinki. This work was conducted in accordance with §12 of the Hamburg hospital law (§12 HmbKHG). The use of anonymized remnant diagnostic samples from patients was approved and informed consent was waived by the ethics committee of the Hamburg Medical Association (PV5626).

## Results

### Analytical performance

**Figure 2:**
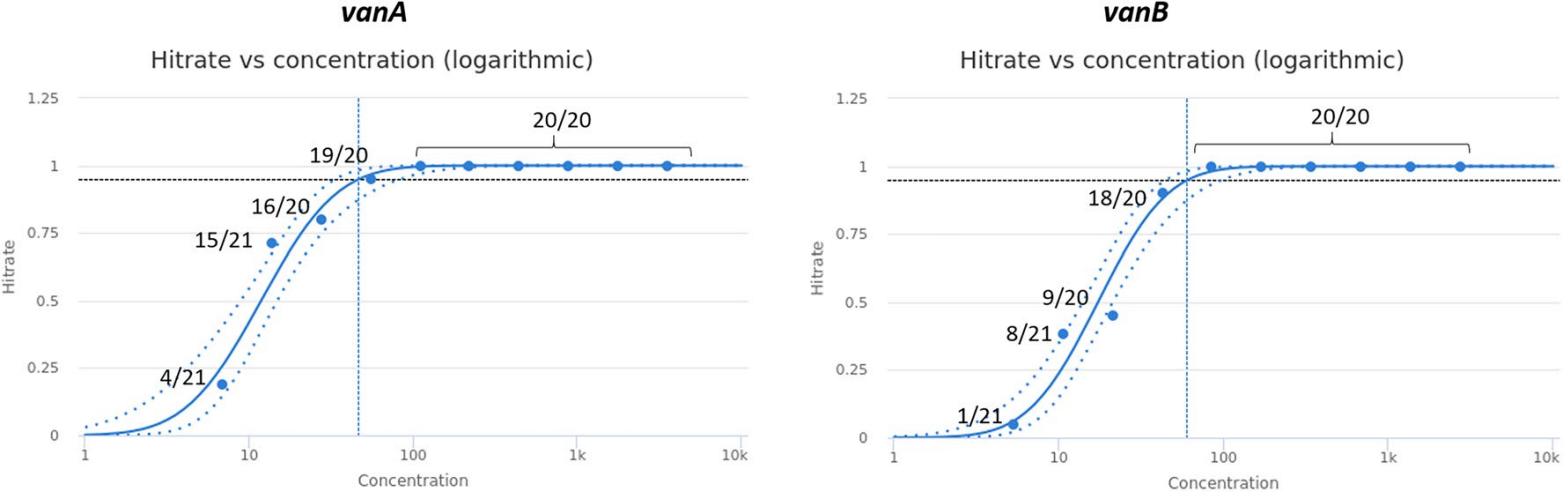
Probit curves of the LoD experiment. Briefly, a twofold dilution series of quantified vanA and vanB standard (quantified by digital PCR) was used to determine the 95% probability of detection (20 repeats per dilution step). Confidence intervals are indicated as dash lines. Hit-rates of each concentration are shown in the graph (see also Table S1).

LoD was determined as 46.9 dcp/ml (CI95%: 33.6–83.3 dcp/ml) for the *vanA*-assay and 60.8 dcp/ml (CI95%: 44.8–97.8 dcp/ml) for the *vanB*-assay by 95% probit analysis (CLSI EP17-A2) using Validation Manager. 1 dcp/ml equals 1.7 colony forming units (CFU)/ml. Probit plots are shown in Fig. 2. Concentrations and hit rates are available in Table S1.

**Figure 3:**
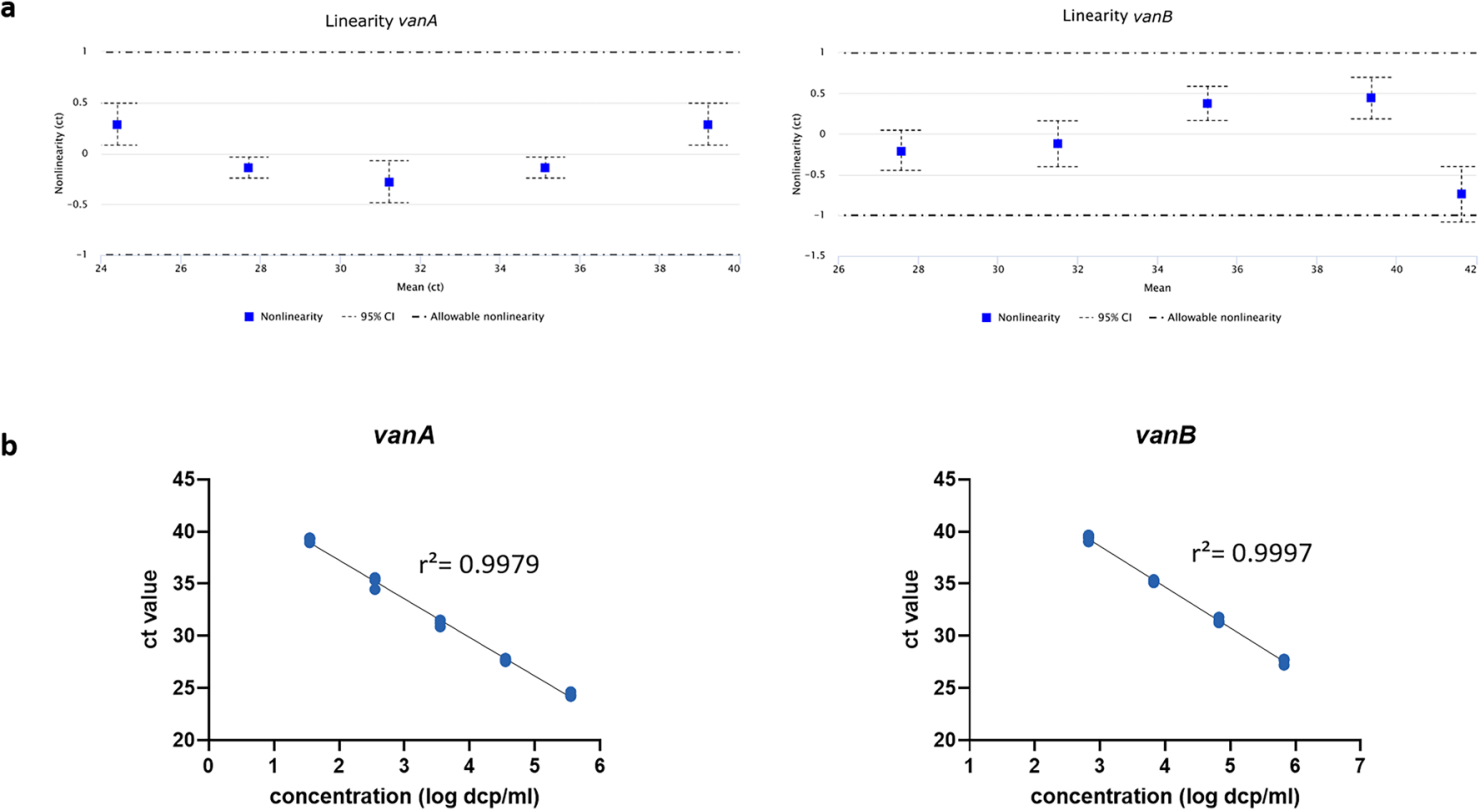
Linearity data for *vanA* (left) and* vanB* (right). Linearity was determined by serial dilution of *vanA* and *vanB* standard material. (**a**) Accepted non-linearity ± 1 ct. (**b**) Linearity fit. *vanA*: slope: − 3.70, r^2^: 0.9979, PCR efficacy: 86.32%; *vanB*: slope: − 3.92, r^2^: 0.9997, PCR efficacy: 87.92%.

The *vanA/B*_UTC assay showed excellent linearity for *vanA* between ct (cycle threshold) 39.2 and ct 24.4 (which equates 3.5 × 10^1^ dcp/ml and 3.5 × 10^5^ dcp/ml) with a pooled SD of 0.343 ct and for *vanB* between ct 39.4 and ct 27.6 (which equates 6.7 × 10^2^ dcp/ml and 6.7 × 10^5^ dcp/ml) with a pooled SD of 0.237 ct (Fig. 3). The PCR efficacy for *vanA* is 86.32% (slope − 3.70, r^2^: 0.9979) and for *vanB* 87.92%* (*slope − 3.92, r^2^: 0.9997).

The intra-run, inter-run and within-laboratory precision ranged between 0.078 ct and 0.710 ct (CV 0.27–1.99%) for *vanA* and between 0.096 ct and 0.535 ct (CV 0.33–1.50%) for *vanB* (Table S2). The equations for quantifications are: *vanA* dcp/ml = 10^(−0.27*ct value) +12.08^ : *vanB* dcp/ml = 10^(−0.27*ct value) +13.45^.

### Inclusivity and exclusivity testing

All external quality assessment samples (n = 28; Instand e.V., Düsseldorf, Germany) were tested correctly (10/10 *vanA* positive, 18/18 *vanA* negative, 7/7 *vanB* positive, 21/21 *vanB* negative) (Fig. S2).

No false positives occurred in the cross-reactivity study using 47 isolates of 36 different common enteric bacteria including other enterococci (e.g., *E. avium, E. gallinarum, E. casseliflavus*) (Fig. S2 and Table S3).

### Clinical evaluation for VRE detection

#### Rectal swab (without pre-culture)

**Figure 4:**
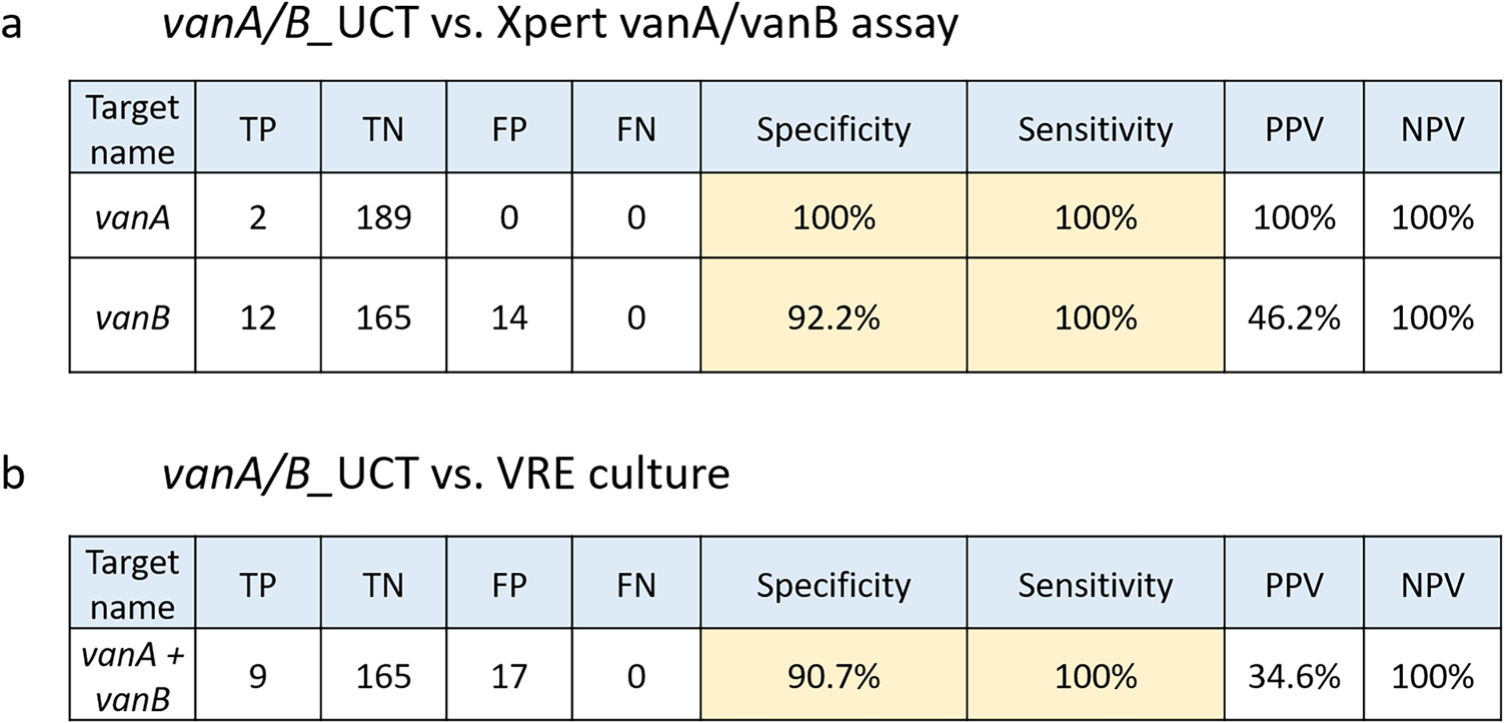
Diagnostic accuracy of the vanA/B_UTC assay compared to Xpert *vanA/vanB* assay (**a**) and culture (**b**). *TP* true positive, *TN* true negative, *FP* false positive, *FN* false negative, *PPV* positive predictive value, *NPV* negative predictive value.

In total, 196 rectal swabs (eSwab) were tested directly without enrichment in broth using the *vanA/B*_UTC assay on the cobas 5800/6800/8800and the CE-IVD Xpert *vanA/vanB* assay systems and its results were compared to culture (enrichment broth + VRE agar). 5/196 samples yielded invalid results in the molecular testing (cobas: 4 samples, 2.0%; Xpert: 1 sample, 0.5%) and were excluded from data analysis. The higher dilution of eSwab samples (1:6) compared to the dilution used in the two-tier approach (1:2, see below) led to a significant decrease of invalid test results (12.8% vs. 2.0%). The cutoff for the *vanA/B*_UTC was set at ct35 for both targets. We first compared the *vanA/B*_UTC assay to the cartridge-based Xpert *vanA/vanB* assay: Two out of 191 samples were correctly identified as *vanA* positive, while no false positives or negatives were detected (sensitivity: 100%, specificity: 100%, Fig. 4a). 12 samples were detected as true *vanB* positive (165 samples true negative, sensitivity: 100%, Fig. 4a) and 14/191 samples were measured as false *vanB* positive using the *vanA/B*_UTC assay (specificity: 92.2%, Fig. 4a). Mean ct levels of true and false positive samples were 28.5 and 32.7, respectively. In addition, a good correlation of ct values between both assays was observed (spearman non parametric test; r = 0.73 p < 0.0001; Fig. S1). Discrepancies between our assay and the VRE Xpert assay may be due to differences in assay set-up, e.g. oligo sequences.

Nine out of 191 samples were VRE positive in culture, which were correctly detected by the new *van*A/B_UTC assay (sensitivity: 100%, Fig. 4b). 17/191 samples were detected as false positives by the qPCR assay, leading to a specificity of 90.7% but a PPV for VRE positivity in culture of only 34.6% (Fig. 4b). Since *vanB* is also present in some anaerobic bacteria the relatively low positive PPV of *vanB* qPCR compared to VRE detection with cultural methods is in line with other studies^25–27^.

#### Two-tier approach (VRE broth followed by qPCR)

**Figure 5:**
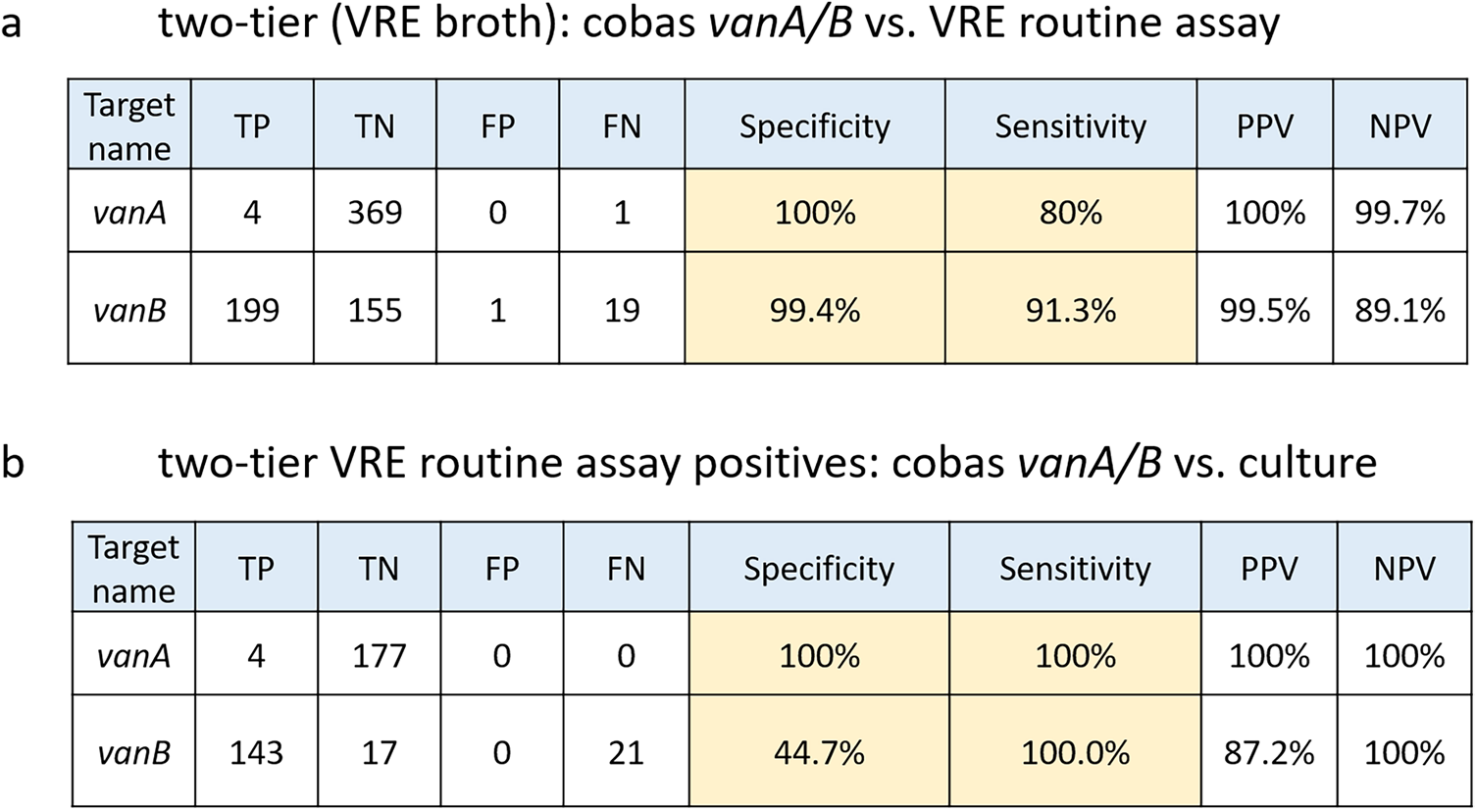
Diagnostic accuracy of the vanA/B_UTC after enrichment in broth compared to our two-tier approach (enrichment and qPCR on a LC480 II) (**a**) and VRE culture (**b**). Culture results were only available in VRE positive samples determined by the two-tier routine assay. *TP* true positive, *TN* true negative, *FP* false positive, *FN* false negative, *PPV* positive predictive value, *NPV* negative predictive value.

An alternative approach to increase PPV for VRE carriage status is the “two-tier” approach, where an enrichment in VRE broth is followed by qPCR for *vanA* and *vanB* detection. Several groups^25,26^ have shown that this approach can significantly increase the PPV for VRE detection in culture. Therefore, we compared the results of the *vanA/vanB*_UTC assay from VRE broth with our routine LDT using a semi-automated workflow (Roche Flow Solution; extraction = MagnaPure96, PCR setup = Hamilton starlet (PSU/PSH); PCR = LightCycler 480 II) and the same primer/probe sets (without modifications) as used in the *vanA/vanB*_UTC assay. The experimental details were previously published^25^. In total 374 routine samples were compared and an overall high agreement was achieved (cutoff *vanA/vanB*_UTC assay: ct 35, cutoff routine VRE assay: ct 32). Briefly, *vanA* was true positive in 4/374 samples and 369/374 samples were correctly detected as *vanA* negative. One sample was discrepant since it was positive in the routine *vanB/vanA* assay, but negative using the new cobas assay and in culture (sensitivity 80%, specificity 100%) (Fig. 5a). *VanB* was true positive in 199/374 samples and true negative in 155/374 samples. One sample was detected as false *vanB* positive and 19 samples were false negative (sensitivity 91.3% and specificity 99.4%) (Fig. 5a).

For 181 samples of this study (ct < 32 in the routine *vanB/vanA* assay) culture results were available. Sensitivity of the *vanA/vanB*_UTC assay compared to culture was 100% for *vanA* (4/181 true positive) and *vanB* (143/181 true negative), and specificity was 100% (177/181 true negative) and 44.7% (17/181 true negative and 21/181 false negative) for *vanA* and *vanB*, respectively, with a PPV of 100% for *vanA* and 87.2% for *vanB* and an NPV of 100% for both targets (Fig. 5b).

#### vanA/vanB quantification

**Figure 6:**
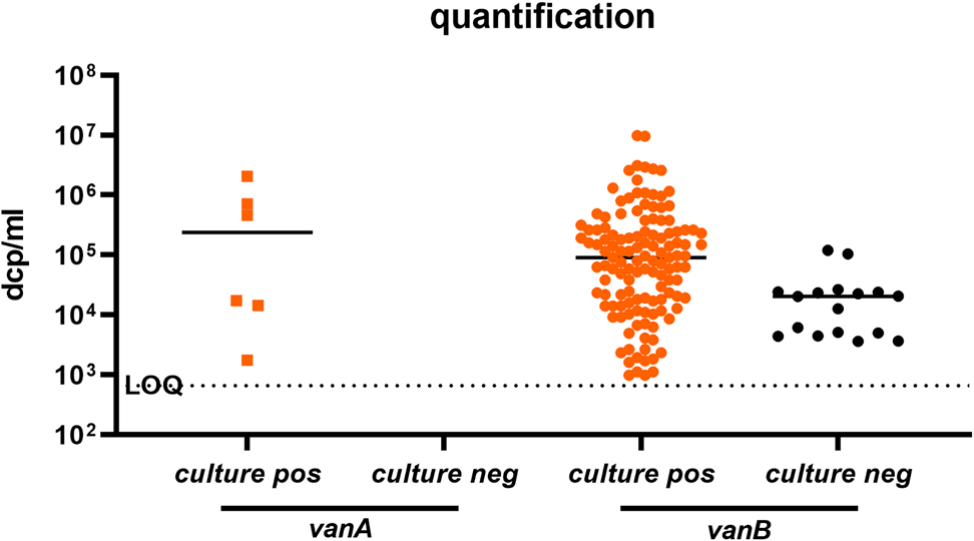
Quantification of *vanA* (square symbols) and *vanB* (dots) in positive samples measured with the vanA/B_UTC assay. Samples that were VRE positive in culture are depicted in orange. Limit of quantification (LOQ) is 660 dcp/ml.

All samples from the cohort “direct swab” (n = 191) and all *vanA/vanB* positive samples from the two-tier cohort (n = 147) were also measured and quantified by *vanA/B*_UCT from swab. In total results from 338 samples were quantitatively analysed (copies/ml) using the quantification formula established from the linearity experiment. For culture positive samples median *vanA* DNA copies/ml was 2.4 × 10^5^ (range 1.7 × 10^3^–2.1 × 10^6^ dcp/ml) and for *vanB* DNA copies/ml 9.1 × 10^4^ dcp/ml (range 9.8 × 10^2^–9.8 × 10^6^ dcp/ml; see Fig. 6). For culture negative *vanB* samples median copies/ml was 2.0 × 10^4^ dcp/ml (range 3.6 × 10^3^–1.2 × 10^5^ dcp/ml).

## Discussion

VRE are one of the leading causes of ICU-acquired bloodstream infections in Europe with high mortality rates^10^. To accelerate screening for and facilitate quantification of VRE in rectal swabs, we adapted a dual target qPCR assay for use on the high throughput, fully-automated cobas 5800/6800/8800 systems (Roche).

The assay demonstrated high sensitivity with LODs of 46.9 digital copies (dcp)/ml for *vanA* and 60.8 dcp/ml for *vanB* and excellent linearity between 4.7 × 10^1^ and 3.5 × 10^5^ dcp/ml (*vanA*) and 6.7 × 10^2^ and 6.7 × 10^5^ dcp/ml (*vanB*). Dilution of rectal swab samples (1:6) prior to detection decreased the amount of invalid results, likely due to a reduction of inhibitory substances present in stool. A spike-in full-process control assay, similar to commercial CE-IVD assays, is already included in the open channel reagents. The *vanA* assay is highly specific (100%), while the *vanB* test resulted in an increased proportion of false positives. The *vanB* resistance determinant is known to be present in gram-positive anaerobes, as well as in enterococci, which likely explains the comparably lower specificity (90.7%) and positive predictive value (PPV) (34.6%) of the *vanB* PCR (cutoff ct35) in this study and in other published assays for VRE screening^22,23,28,29^. The use of enterococcus enrichment broth, which facilitates the outgrowth of enterococci over anaerobes, improved the PPV of the *vanB* screening to 87.2%, which is in line with results from other studies^26^.

Overall, evidence to support VRE screening of every hospitalized patient is heterogeneous. While in one study, single room precautions led to a significant reduction in transmission events in haematological/oncological wards^30^, another study could not show a significant effect^31^. Possible benefits in transmission prevention must be weighed against negative effects of contact precautions on the individual patient’s care^17,32,33^. Better knowledge of risk factors for transmission and infection will help to further improve VRE-related infection prevention and control measures in hospitals. For example, antibiotic therapy, especially with anti-anaerobic activity, is associated with an increase in VRE density in stool^34^. At the same time, antibiotic therapy has also been shown to increase the risk of VRE infection in hospitalized patients^3,35–40^. It can be hypothesized that overgrowth of VRE in the gut may contribute to an increased risk of infection, for example through contamination of urinary catheters or central venous catheters. Our assay enables the quantification of *vanA/vanB* determinants directly from rectal swab samples. We could show that some patients had levels up to 10^7^ dcp/ml *vanA* or *vanB* DNA/ml (rectal swab), indicating massive VRE colonisation. In the future, it may be of interest to investigate the pathophysiological mechanisms of VRE infection at the intersection of VRE density, changes in the gut microbiota and patient-specific risk factors.

In conclusion, we provided a technical performance evaluation for a lab-developed duplex qPCR-assay for *vanA* and *vanB* detection on the cobas 5800/6800/8800 high-throughput systems. The assay enables rapid detection and quantification of VRE directly from rectal swab samples or VRE enrichment broth in a two-tier approach and may support future clinical studies assessing the impact of bacterial load on risk of infection and transmission.

### Supplementary Information

Supplementary Information.

## Data Availability

The datasets generated and analyzed during the current study are available from the corresponding author on reasonable request.
