# Supplementary material for: Adaptation and validation of a quantitative vanA/vanB DNA screening assay on a high-throughput PCR system
Source: Sci Rep. 2024 Feb 12;14:3523. doi: 10.1038/s41598-024-54037-5 (PMC10861526; doi:10.1038/s41598-024-54037-5)
Supplement: Supplementary file 1 — Supplementary Information. [file 41598_2024_54037_MOESM1_ESM.docx]

**Adaptation and validation of a quantitative *vanA*/*vanB* DNA screening assay on a high-throughput PCR system**

Katja Giersch^1#^, Konstantin Tanida^1#^, Anna Both^1^, Dominik Nörz^1^, Denise Heim^1^, Holger Rohde^1^, Martin Aepfelbacher^1^, Marc Lütgehetmann^1^

1. Institute of Medical Microbiology, Virology and Hygiene, University Medical Centre Hamburg-Eppendorf (UKE), Hamburg, Germany

# shared authorship

| *vanA* | | | |  | *vanB* | | | |
| --- | --- | --- | --- | --- | --- | --- | --- | --- |
| **Concentration (dcp/ml)** | **Positive results** | **Hitrate** | **Average Ct** |  | **Concentration (dcp/ml)** | **Positive results** | **Hitrate** | **Average Ct** |
| 3542.50 | 20 / 20 | 1 | 31.0 |  | 2736.00 | 20 / 20 | 1 | 37.2 |
| 1771.25 | 20 / 20 | 1 | 32.3 |  | 1368.00 | 20 / 20 | 1 | 38.7 |
| 885.63 | 20 / 20 | 1 | 33.6 |  | 684.00 | 20 / 20 | 1 | 40.5 |
| 442.81 | 20 / 20 | 1 | 34.2 |  | 342.00 | 20 / 20 | 1 | 40.7 |
| 221.41 | 20 / 20 | 1 | 35.6 |  | 171.00 | 20 / 20 | 1 | 42.3 |
| 110.70 | 20 / 20 | 1 | 36.1 |  | 85.50 | 20 / 20 | 1 | 41.9 |
| 55.35 | 19 / 20 | 0.95 | 36.7 |  | 42.75 | 18 / 20 | 0.9 | 43.3 |
| 27.68 | 16 / 20 | 0.8 | 37.0 |  | 21.38 | 9 / 20 | 0.45 | 44.0 |
| 13.84 | 15 / 21 | 0.714 | 39.0 |  | 10.69 | 8 /20 | 0.381 | 44.1 |
| 6.92 | 4 / 21 | 0.19 | 38.3 |  | 5.34 | 1 / 20 | 0.0476 | 43.4 |

***Table S1****. LoDs were determined by serial dilution of a vanA and vanB standard (quantified by digital PCR). dcp = digital copies, ct = cycle threshold*

***
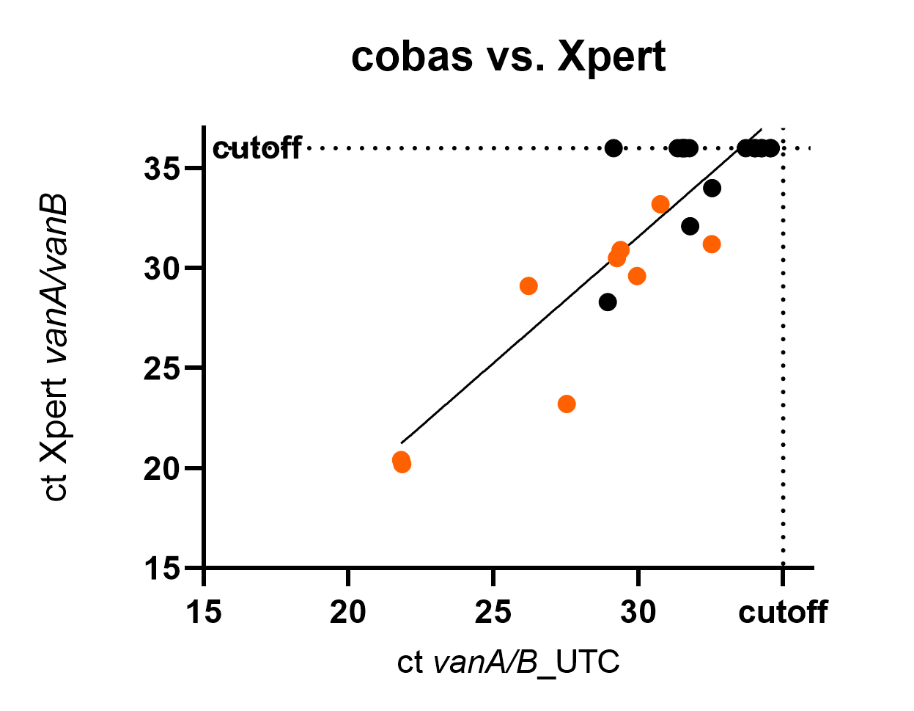
***

***Figure S1.*** *Correlation of ct values between the new vanA/B_UTC assay and the Xpert vanA/vanB assay. Samples that were VRE positive in culture are depicted in orange.*

|  | | | | **Intra-run** | | **Inter-run** | | **within-laboratory** | |
| --- | --- | --- | --- | --- | --- | --- | --- | --- | --- |
| **Analyte** | **Sample** | **Mean** | **N** | **SD (ct)** | **CV (%)** | **SD (ct)** | **CV (%)** | **SD (ct)** | **CV (%)** |
| VRE: v*anA* | vre_step1 | 28.4 | 9 | 0.0778 (0.0501 – 0.171) | 0.274% (0.176% – 0.602%) | 0.389 (0.201 – 2.55) | 1.37% (0.708% – 8.97%) | 0.397 (0.209 – 2.3) | 1.4% (0.735% – 8.1%) |
|  | vre_step2 | 32.2 | 9 | 0.236  (0.152 – 0.519) | 0.732% (0.472% – 1.61%) | 0.281 (0.133 – 4.23) | 0.872% (0.412% – 13.1%) | 0.367 (0.216 – 1.14) | 1.14% (0.67% – 3.54%) |
|  | vre_step3 | 35.7 | 9 | 0.582  (0.375 – 1.28) | 1.63% (1.05% – 3.59%) | 0.407 (0.166 – 68.6) | 1.14% (0.464% – 192%) | 0.71 (0.449 – 1.66) | 1.99% (1.26% – 4.66%) |
| VRE: *vanB* | vre_step1 | 29.1 | 9 | 0.096 (0.0619 – 0.211) | 0.33% (0.213% – 0.727%) | 0.284 (0.146 – 2.02) | 0.977% (0.5% – 6.95%) | 0.3 (0.161 – 1.54) | 1.03% (0.553% – 5.28%) |
|  | vre_step2 | 32.6 | 9 | 0.147 (0.0945 – 0.323) | 0.45% (0.29% – 0.991%) | 0.26  (0.13 – 2.34) | 0.799% (0.398% – 7.2%) | 0.299 (0.167 – 1.2) | 0.917% (0.513% – 3.68%) |
|  | vre_step3 | 35.7 | 9 | 0.356  (0.23 – 0.784) | 0.997% (0.642% – 2.19%) | 0.4  (0.187 – 6.84) | 1.12% (0.523% – 19.1%) | 0.535 (0.318 – 1.6) | 1.5% (0.889% – 4.48%) |

***Table S2****. Precision of the vanA/B_UTC assay. Intra-run, inter-run and within-laboratory precision (ANOVA) was determined using vanA and vanB reference material in three 1:10 dilution steps in triplicates on three consecutive days. SD: standard deviation (in ct), CV: coefficient of variation (in %).*


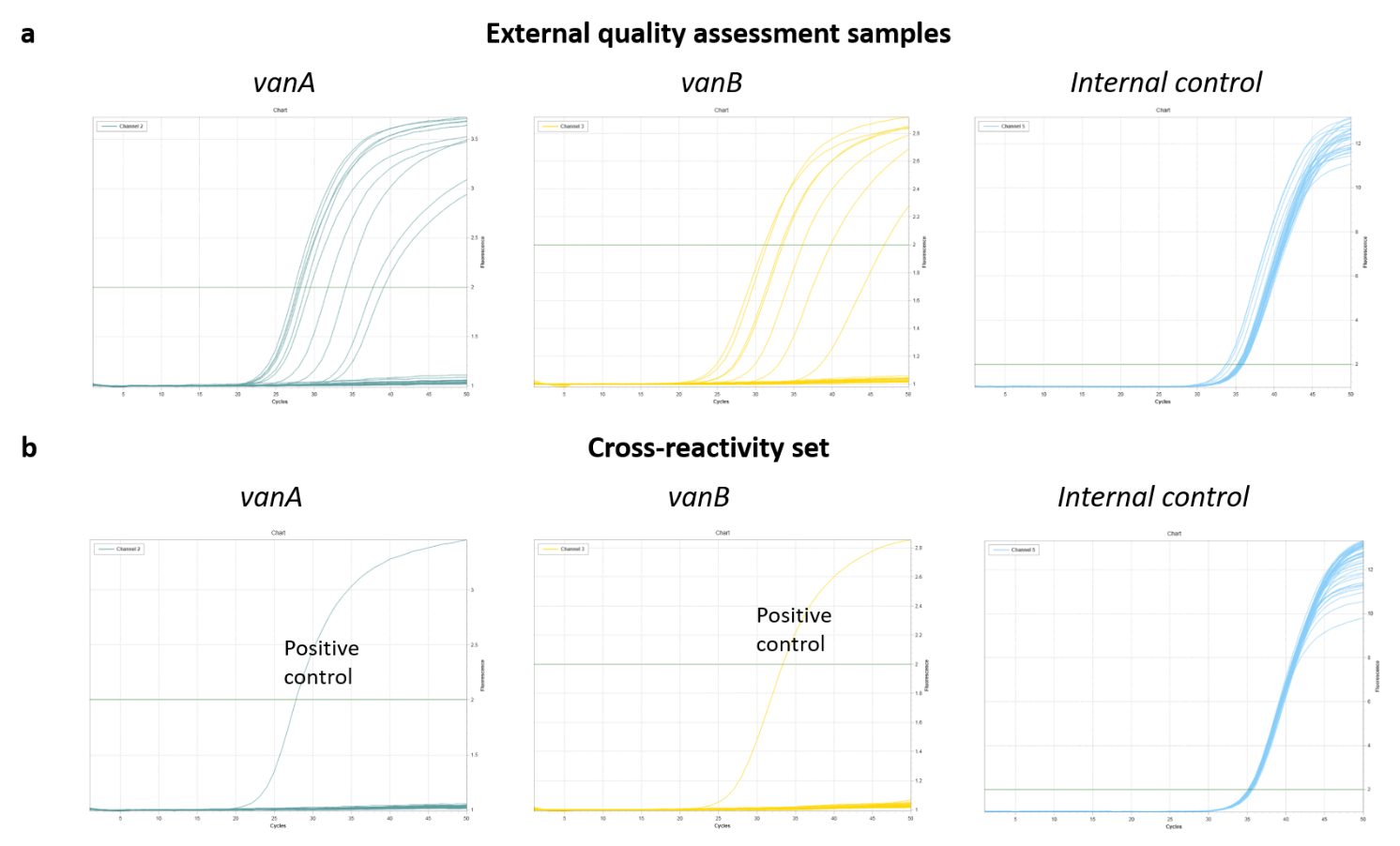


***Figure S2.*** *qPCR amplification curves of vanA, vanB and the internal control of the Inclusivity and exclusivity testing using the vanA/B_UTC assay.* ***a****) External quality assessment set: 10/10 vanA positive, 18/18 vanA negative, 7/7 vanB positive, 21/21 vanB negative.* ***b****) Cross-reactivity study: n= 47 plus one positive control.*

| **Bacteria** | **Isolates tested** | ***vanA*** | ***vanB*** |
| --- | --- | --- | --- |
| *Achromobacter xylosoxidans* | 1 | negative | negative |
| *Aerococcus urinae* | 1 | negative | negative |
| *Bacteroides fragilis* | 1 | negative | negative |
| *Citrobacter freundii* | 1 | negative | negative |
| *Corynebacterium amycolatum* | 1 | negative | negative |
| *Corynebacterium simulans* | 1 | negative | negative |
| *Enterobacter cloacae complex* | 1 | negative | negative |
| *Enterococcus avium* | 5 | negative | negative |
| *Enterococcus casseliflavus* | 2 | negative | negative |
| *Enterococcus faecalis* | 5 | negative | negative |
| *Enterococcus faecium* | 3 | negative | negative |
| *Enterococcus gallinarum* | 2 | negative | negative |
| *Escherichia coli* | 1 | negative | negative |
| *Fusobacterium necrophorum* | 1 | negative | negative |
| *Klebsiella oxytoca* | 1 | negative | negative |
| *Klebsiella pneumonia* | 1 | negative | negative |
| *Lactobacillus gasseri* | 1 | negative | negative |
| *Lactobacillus paracasei* | 1 | negative | negative |
| *Lactobacillus rhamnosus* | 1 | negative | negative |
| *Listeria monocytogenes* | 1 | negative | negative |
| *Micrococcus luteus* | 1 | negative | negative |
| *Morganella morganii* | 1 | negative | negative |
| *Staphylococcus aureus* (MRSA) | 1 | negative | negative |
| *Parabacteroides merdae* | 1 | negative | negative |
| *Proteus mirabilis* | 1 | negative | negative |
| *Providencia rettgeri* | 1 | negative | negative |
| *Pseudoglutamicibacter cumminsii* | 1 | negative | negative |
| *Pseudomonas aeruginosa* | 1 | negative | negative |
| *Streptococcus agalactiae* | 1 | negative | negative |
| *Serratia marcescens* | 1 | negative | negative |
| *Staphylococcus aureus* | 1 | negative | negative |
| *Streptococcus mitis* | 1 | negative | negative |
| *Streptococcus pneumoniae* | 1 | negative | negative |
| *Streptococcus pyogenes* | 1 | negative | negative |
| *Streptococcus sanguinis* | 1 | negative | negative |
| *Veilonella parvula* | 1 | negative | Negative |

***Table S3.*** *47 isolates of 36 different common enteric bacteria were used for the exclusivity set. No false positives occurred.* MRSA = methicillin-resistant *Staphylococcus* *aureus*
